# Thermal acclimation of photosynthesis and respiration of southern and northern white spruce seed sources tested along a regional climatic gradient indicates limited potential to cope with temperature warming

**DOI:** 10.1093/aob/mcx174

**Published:** 2017-12-29

**Authors:** Lahcen Benomar, Mohammed S Lamhamedi, Steeve Pepin, André Rainville, Marie-Claude Lambert, Hank A Margolis, Jean Bousquet, Jean Beaulieu

**Affiliations:** 1 Centre d’étude de la forêt, Faculté de foresterie, de géographie et de géomatique, Pavillon Abitibi Price, Université Laval, Québec, Canada; 2 Direction de la recherche forestière, ministère des Forêts, de la Faune et des Parcs, 2700 rue Einstein, Québec, Canada; 3 Faculté des sciences de l’agriculture et de l’alimentation, Pavillon de l’Envirotron, Université Laval, Québec, Canada

**Keywords:** *Picea glauca*, thermal acclimation, climate change, photosynthesis, respiration, assisted migration, local adaptation, temperature, maximum rate of carboxylation, maximum electron transport rate, mesophyll conductance

## Abstract

**Background and Aims:**

Knowledge of thermal acclimation of physiological processes of boreal tree species is necessary to determine their ability to adapt to predicted global warming and reduce the uncertainty around the anticipated feedbacks of forest ecosystems and global carbon cycle to climate change. The objective of this work was to examine the extent of thermal acclimation of net photosynthesis (*A*_n_) and dark respiration (*R*_d_) of two distant white spruce (*Picea glauca*) seed sources (from south and north of the commerial forest zone in Québec) in response to latitudinal and seasonal variations in growing conditions.

**Methods:**

The temperature responses of *A*_n_, its biochemical and biophysical limitations, and *R*_d_ were measured in 1-year-old needles of seedlings from the seed sources growing in eight forest plantations along a regional thermal gradient of 5.5 °C in Québec, Canada.

**Key Results:**

The average optimum temperature (*T*_opt_) for *A*_n_ was 19 ± 1.2 °C and was similar among seed sources and plantation sites along the thermal gradient. Net photosynthesis at *T*_opt_ (*A*_opt_) varied significantly among plantation sites and was quadratically related to the mean July temperature (MJT) of plantation sites. *T*_opt_ for mesophyll conductance, maximum electron transport rate and maximum rate of carboxylation were 28, 22 and 30 °C, respectively. Basal respiration rate (*R*_d_ at 10 °C) was linearly and negatively associated with MJT. *Q*_10_ of *R*_d_ (the rate of change in *R*_d_ with a 10 °C increase in temperature) did not show any significant relationship with MJT and averaged 1.5 ± 0.1. The two seed sources were similar in their thermal responses to latitudinal and seasonal variations in growing conditions.

**Conclusions:**

The results showed moderate thermal acclimation of respiration and no evidence for thermal acclimation of photosynthesis or local genetic adaptation for traits related to thermal acclimation. Therefore, growth of local white spruces may decline in future climates.

## INTRODUCTION

In the large boreal forest of Canada, global warming should be leading to an increase in average daily temperature of at least 2 °C (scenario B1) by 2050 (IPCC, 2014) and to rises in the frequency and duration of summertime episodes of extreme heat waves and drought (Romero-Lankao *et al*., 2014). It is unclear today whether changes in average climatic factors or extreme events will be the main drivers of species responses to climate change. Downregulation of net CO_2_ uptake as a result of global warming may reduce species fitness and ecosystem feedback on the global carbon cycle (Sage *et al.*, 2008). It is important to understand how physiological processes involved in photosynthetic and respiration rates will respond to future climatic regimes in order to (1) accurately predict climate change effect on carbon uptake at different scales (Way and Yamori, 2014), (2) reduce the uncertainty around the anticipated feedbacks of forest ecosystems, and global carbon cycle to climate change (Niu *et al.*, 2012; Girardin *et al.*, 2016) and (3) determine the intrinsic acclimation and genetic abilities of tree species to adapt to climate change in the short and longer terms (Bigras, 2000; Way and Sage, 2008*b*; Gunderson *et al.*, 2010). Thermal acclimation of both dark respiration (*R*_d_) and net photosynthetic rate (*A*_n_) through biochemical, biophysical and structural adjustments may help plants to maintain a positive carbon balance in warming conditions (Medlyn *et al.*, 2002; Atkin *et al.*, 2005; Sage *et al.*, 2008; Way and Yamori, 2014). However, the extent to which thermal acclimation may help boreal conifer species to cope with global warming remains poorly understood. Currently, few reports show a lack, or very limited thermal acclimation, of *A*_n_ for boreal tree species (Way and Sage, 2008*a, b*; Dillaway and Kruger, 2010; Ow *et al.*, 2010; Silim *et al.*, 2010; Zhang *et al.*, 2015). Conversely, it has been reported that boreal tree species may show a moderate to strong thermal acclimation of *R*_d_ in response to experimental warming (Gunderson *et al.*, 2000; Ow *et al.*, 2008; Way and Sage, 2008*b*; Silim *et al.*, 2010; Zhang *et al.*, 2015; Reich *et al.*, 2016), or following a thermal latitudinal gradient (Tjoelker *et al.*, 2009; Dillaway and Kruger, 2011).

Thermal acclimation of CO_2_ exchange has been mostly investigated in controlled conditions using static day–night temperature treatments or from seasonal variation in CO_2_ exchange in response to the seasonal courses of temperature (Hikosaka *et al.*, 2006; Way and Yamori, 2014; Yamori *et al.*, 2014). Although these studies helped increase our understanding of the processes involved in thermal acclimation, their results cannot realistically be used to infer thermal responses in natural conditions, and consequently to better evaluate the quantitative aspects of tree responses to global warming. The main reason for this is the lack of representation of diurnal and daily temperature variations during the growing season, which are especially important in boreal regions. In addition, it is unclear whether or not thermal acclimation of *A*_n_ to temporal variation in temperature during the growing season and spatial variation in temperature along a climate gradient may result from similar physiological adjustments. In fact, the predominant role of photoperiod in the regulation of the seasonal pattern of photosynthetic rate, also known as the phenology of photosynthesis, is still largely controversial for tree species (Busch *et al.*, 2007; Bauerle *et al.*, 2012; Stinziano and Way, 2017).

Thermal acclimation of both *A*_n_ and *R*_d_varies widely among tree species depending on their thermal environment (Aitken *et al.*, 2008; Dillaway and Kruger, 2010; Way and Yamori, 2014; Reich *et al.*, 2016). Thermal acclimation of *A*_n_ can improve or at least maintain plant photosynthetic performance when the growth temperature regime shifts from cold to warm through adjustments of one or more photosynthetic components (Way and Yamori, 2014). This may occur via (1) the shift in the thermal optimum of *A*_n_ (*T*_opt_) towards the warm growing temperature, (2) the increase or maintenance of the photosynthetic rate at *T*_opt_ (*A*_opt_) in the new growing temperature conditions, (3) the shift in both *A*_opt_ and *T*_opt_ (Way and Yamori, 2014), or (4) the increase or maintenance of the photosynthetic rate with respect to growth temperature (*A*_growth_). The mechanisms involved in thermal acclimation of photosynthesis include adjustment in (1) thermal responses of both maximum rate of carboxylation (*V*_cmax_) and maximum electron transport rate (*J*_max_) (activation and deactivation energy), (2) basal *V*_cmax_ measured at a reference temperature of 25 °C (*V*_cmax25_) and *J*_max25_, (3) the ratio of *J*_max25_ to *V*_cmax25_, and (4) thermal responses of mesophyll (*g*_m_) and stomatal conductance (*g*_s_) (Kattge and Knorr, 2007; Warren, 2008; Silim *et al.*, 2010; Way and Yamori, 2014).

Thermal acclimation of *R*_d_ in response to the increase in temperature may occur via (i) a downregulation of the basal rate of *R*_d_ (so-called type II acclimation), (2) a decrease in *Q*_10_ (rise in *R*_d_ with a 10 °C increase in temperature) (type I acclimation), or (3) a combination of both types (Atkin and Tjoelker, 2003; Atkin *et al.*, 2005; Way and Yamori, 2014; Reich *et al.*, 2016).

Mineral nutrition is one of several physiological attributes that contribute to the improvement of survival, growth and physiology of tree seedlings after outplanting (Margolis and Brand, 1990). Several studies showed that net photosynthesis, dark respiration and survival were closely related to nitrogen levels in needles (Lamhamedi and Bernier, 1994; Tjoelker *et al.*, 1999; Poorter *et al.*, 2009). Needle nitrogen concentration (*N*_mass_) varies with site climatic conditions, including temperature (Friend *et al.*, 1989). However, little is known about the role of *N*_mass_ in thermal acclimation of *A*_n_ and *R*_d_. For instance, Tjoelker *et al.* (2009) showed that both type I and II acclimation of *R*_d_ were unrelated to *N*_mass_ in *Pinus banksiana*.

Clinal variation in growth and other functional traits as a result of local genetic adaptation to climate of origin has been reported for several boreal tree species (Li *et al.*, 1997; Andalo *et al.*, 2005; Aitken *et al.*, 2008; Benomar *et al.*, 2016). However, little evidence exists regarding the genetic differentiation in the thermal acclimation capacity and its involvement in growth clinal variation. Recently, Drake *et al.* (2017) found similar thermal responses (both *A*_n_ and *R*_d_) in response to an experimental warming (under controlled conditions) of three seed sources of *Eucalyptus tereticornis* despite a large geographical distance among them and a 13 °C difference in mean annual temperature at seed origin. Similar results were reported for *Pinus banksiana* (Tjoelker *et al.*, 2009). In contrast, Ishikawa *et al.* (2007) showed intraspecific variation in thermal acclimation of *A*_n_ among *Plantago asiatica* populations, which was related to the capacity of adjustement of the *J*_max25_ to *V*_cmax25_ ratio.

White spruce (*Picea glauca*) is one of the ecologically and commercially most important conifer species of the boreal forest of Canada (Beaulieu *et al.*, 2009). It is the subject of large reforestation efforts and several intensive breeding programmes in Canada (Mullin *et al*., 2011). It has been shown not to be optimally adapted to local climate conditions in relation to recent temperature warming (e.g. Andalo *et al.*, 2005). But to our knowledge, no one has investigated the temperature response of both photosynthesis and respiration to determine the thermal acclimation capacity and potential adaptive differences among seed sources from geographically distant regions, which could affect the productivity of forest ecosystems and assisted migration strategies. The objectives of this study were (1) to evaluate the thermal acclimation of photosynthesis and respiration of two geographically distant white spruce seed sources in response to short-term variation in climatic conditions during a growing season and to long-term variation in growing conditions along a regional thermal gradient of 5.5 °C, and (2) to assess the involvement of morphological, biochemical and biophysical processes in the temperature response of photosynthesis.

## MATERIALS AND METHODS

### Genetic material and planting sites

**Fig. 1.**
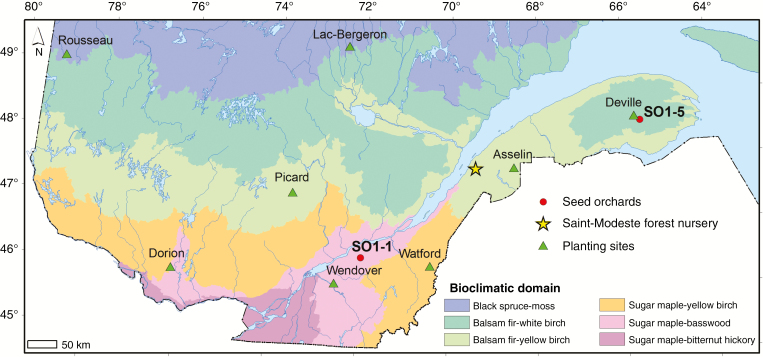
Location of the eight forest plantation sites and the two white spruce seed sources tested in the study. SO1-1, southern seed source; SO1-5, northern seed source.

The two white spruce seed sources used in this study were chosen to represent the south and north of the large commercial forest zone in Québec. They came from two first-generation seed orchards (SO1-1 and SO1-5) commonly used for reforestation in Québec, Canada (Fig. 1), distant from each other by 550 km and 2.2° of latitude (Table 1). They are clonal seed orchards made of grafted plus-trees that were selected in local natural stands. They each pertain to one of the two broadly defined white spruce breeding zones in Québec, Canada, which differ mostly in latitude and associated mean annual temperature (Li *et al.*, 1997). Orchard SO1-1 represents the southern seed source and SO1-5 the northern seed source (Fig. 1). Open-pollinated seeds were collected in each seed orchard for two consecutive years (2008 and 2009) and mixed, making up one seedlot per seed orchard. Seedling production was conducted under nursery conditions at the Pépinière forestière of Saint-Modeste (Québec, Canada, 47°50′ N, 69°30′ W) and subjected to standard cultural practices during two consecutive growing seasons (Lamhamedi *et al.*, 2006; Villeneuve *et al.*, 2016).

**Table 1.** Location and climatic conditions of the two white spruce seed sources used in this study and the eight forest plantation sites

	Location	Bioclimatic domain*	Latitude	Longitude	Elevation	GDD5	MAT	MJT	MGST	TGSP
			(°N)	(°W)	(m)		(°C)	(°C)	(°C)	(mm)
Seed source										
Northern (SO1-5)	Robidoux	BF-WB	48.55	65.59	270	1298	2.6	16.7	12.9	500
Southern (SO1-1)	St-Cyrille	SM-B	46.39	71.94	116	1708	4.2	18.7	14.9	539
Plantation site^†^										
Lac Bergeron	Dolbeau	S-M	49.65	72.41	434	1347	0.3	16.3	13.8	520
Rousseau	Villebois	S-M	49.15	79.20	291	1516	1.4	17.3	14.9	433
Deville	Robidoux	BF-WB	48.62	65.72	530	1179	1.5	16.9	12.4	536
Asselin	Squatec	BF-YB	47.84	68.52	370	1500	2.5	18.7	14.0	564
Picard	La Tuque	BF-YB	47.34	73.57	460	1397	1.5	16.2	14.4	488
Watford	Ste-Rose	SM-YB	46.30	70.40	385	1644	3.8	18.6	13.9	690
Dorion	Maniwaki	SM-B	46.05	76.26	201	1933	4.8	19.3	16.6	465
Wendover	St-Cyrille	SM-B	45.98	72.52	68	2153	5.8	20.4	15.9	650

GDD5, number of growing degree-days >5 °C; MAT, mean annual temperature; MJT, mean July temperature; MGST, mean growing season temperature; TGSP, total growing season precipitation.

*SM-B, sugar maple–basswood domain; SM-YB, sugar maple–yellow birch domain; BF-YB, balsam fir–yellow birch domain; BF-WB, balsam fir–white birch domain; S-M, spruce–moss domain.

^†^Data for plantation sites are means for years corresponding to the first and second growing seasons.

Eight forest sites representative of the white spruce commercial zone in Québec, Canada, were selected for this study (Table 1, Fig. 1). They cover latitudinal and longitudinal transects of 4.6° and 14°, respectively. Plantations were established in the spring of 2013 for the localities of Watford, Asselin and Deville, in the spring of 2014 for the localities of Wendover, Picard and Lac Bergeron, and in the spring of 2015 for the localities of Dorion and Rousseau. The Watford and Wendover sites were formerly occupied by black spruce (*Picea mariana*) plantations that were harvested in 2012. All other sites, covered by natural forest stands, were also harvested in 2012. Physico-chemical soil properties of plantation sites assessed during the second growing season after plantation are provided in Supplementary Data Table S1.

Two-year-old seedlings were planted at densities of 2000 stems per hectare. Seedling height and root collar diameter (mean ± s.d.) before planting were 38.3 ± 3.6 and 6.9 ± 0.9 mm respectively. Before planting, seedling nutrient concentrations (stem, needles and roots) were assessed using three composite samples (five seedlings/composite sample) per seed orchard (data not shown). The results confirmed that the seedlings met the 28 morphophysiological standards used for white spruce containerized seedling production in Québec (Veilleux *et al.*, 2010).

Seedlings were planted at each site following a randomized complete block design with four blocks. Each seed source was randomly assigned to a plot within a block. The size of each plot was about 730 m^2^ and contained 144 trees (12 × 12 rows of trees) in which only the 64 interior trees were considered for analyses, leaving 4 × 4 rows of border trees as a buffer zone. The total number of planted seedlings was 9216, corresponding to 144 seedlings × 4 blocks × 2 seed sources × 8 sites.

During the first and second growing seasons after plantation in the field, weed competition was controlled mechanically at all sites, except for Deville and Lac Bergeron, where weedy vegetation was scarce.

### Climatic data along the latitudinal gradient

For each plantation site, climatic data (Table 1) were interpolated from data collected from 2013–16 in nearby weather stations using the BioSIM software (Régnière and St-Amant, 2007). Furthermore, permanent weather stations were installed in the Watford, Asselin and Deville sites since 2013, with collected data used to confirm the accuracy of BioSIM data. Climate conditions varied considerably among the sites during the two years of the experiment (Table 1). Mean annual temperature (MAT) was highly influenced by site location. It differed by 5.5 °C between the coolest and the warmest sites. Total growing season precipitation (TGSP) decreased with the longitude of the plantation sites (Table 1).

Natural climatic gradients usually consider geographical and environmental factors such as latitude, longitude, altitude, annual and seasonal mean temperature and precipitation. In the present study, the term ‘climatic gradient’ is used as a simplifier and refers to the annual and seasonal mean temperature gradient, given that the focus of our study was mostly related to change in temperature regime, which was the most variable climatic factor among the plantation sites (Table 1).

### Growth

Seedling height (*H*_2_) and survival were measured at the end of the second growing season on plantation sites (mid-October) on the 64 central trees in each plot, for a total of 4096 trees (64 plants × 4 blocks × 2 seed source × 8 sites).

### Gas exchange measurements

Gas exchange measurements were made with two portable open-path gas-exchange systems (Li-6400, Li-Cor, Lincoln, NE, USA), equipped with a Lighted Conifer Chamber (6400-22L, Li-Cor, Lincoln, NE, USA) and the expanded Temperature Control Kit (6400-88, Li-Cor, Lincoln, NE, USA). Temperature (*T*) response curves of shoot respiration (*R*_d_*–T*) and net photosynthesis (*A*_n_*–T*) were generated for one randomly selected plant per plot at each plantation site (2 seed sources × 3 blocks × 8 sites = 48). Both *A*_n_*–T* and *R*_d_*–T* curves were assessed using 1-year-old needles of the uppermost lateral shoot. The measurements were carried out in June 2015 in the plantations of Watford, Asselin, Deville, Wendover and Lac Bergeron, and in June 2016 in the plantations of Watford, Dorion, Picard and Rousseau. At the Watford, Asselin, Deville and Picard sites, plants were at their third growing season, whereas those at the remaining sites were at their second growing season. The results obtained in Watford during the third and fourth growing seasons were quite similar for thermal acclimation-related traits, which suggested the absence of an age effect on the observed patterns.

The expanded temperature control kit, which contains two water jackets through which water is circulated using a submersible pump, was used to make measurements at low temperatures (10 and 15 °C). The water channels were connected to a water bath and the temperature was controlled by adding ice water. For temperatures above 25 °C, the seedling and the entire gas-exchange system were covered by a plastic tent (hand-made closed chamber using transparent plastic and wood pickets, and measuring 1 m × 1 m 1.5 m). The temperature within the plastic tent was brought to the desired temperature, i.e. between 30 and 40 °C, using a portable 1500 W ceramic heater (CZ448, Comfort Zone, Pottsville, PA, USA). This made it possible to maintain the difference between the ambient air and that within the conifer chamber below 2 °C, and to prevent water condensation in the exhaust tube or in the cuvette. In fact, because of the large volume of the conifer chambers, there is an important issue of water condensation when the temperature in the chamber is warmer than that of the entering air.

Cuvette temperature during measurement varied systematically from low (10 °C) to high (40 °C) by 5 °C increments, and measurements were taken systematically in order from low to high temperature. Seedlings were allowed to acclimate for at least 20 min for each new temperature, before recording data. The attachment points of the shoot to the cuvette walls were taped (adhesive putty) to avoid leaks into and out of the cuvette. Photosynthesis (*A*_n_) was measured under saturating photosynthetically active radiation (PAR = 1000 µmol m^−2^ s^−1^) and at 400 µmol mol^−1^ of CO_2_. Following *A*_n_ measurement, the light source was turned off and *R*_d_ was recorded after at least 15 min of darkness. The vapor pressure deficit (VPD) in the conifer chamber ranged from 0.6 to 3.6 kPa. During measurement, the entering air passed through the drierite column (anhydrous calcium sulphate) to maintain the air humidity (RH) below 75 % at the lower temperature (10 °C). At higher temperatures, a minimum RH of 45 % was maintained by adding water vapour to the air inside the plastic chamber. For each sample, data required to build *A*_n_–*T* and *R*_d_–*T* curves were collected generally within 4–5 h.

Seasonal patterns of *R*_d_–*T* and *A*_n_–*T* were determined at the Watford site during the growing season of 2016. The measurements were taken each month from May to October. The measurements were performed on the same shoot from six seedlings that were different from those used previously for *A–C*_i_*–T* (see below) and those measured in 2015. For practical reasons, we chose the tallest seedlings (with a long shoot) within each subplot. At the end of measurements, in October, the shoots did not show any visible damage and loss of initial leaf area.

### CO_2_ response of net photosynthesis at different temperatures (*A–C*_i_–*T*)

During the active growing season, *A–C*_i_ response curves at temperature 10, 15, 20, 25, 30, 35 and 40 °C were generated in July 2015 at the Watford and Deville plantation sites (respectively the easiest to access among southern and northern sites) and again in July 2016 at the Watford plantation site. The *A*–*C*_i_ response curve measurements were taken after 20 min of steady-state conditions at the ambient atmospheric CO_2_ partial pressure (*C*_a_ = 400 µmol mol^−1^) and at saturated photosynthetic active radiation (PAR = 1000 µmol m^−2^ s^−1^). Thereafter, for a given temperature, the reference CO_2_ (*C*_a_) was changed in the following order: 400, 350, 300, 250, 200, 100, 50, 400, 500, 600, 700, 800, 1000, 1200, 1400 and 1500 µmol mol^−1^. Values were recorded based on the stability of photosynthesis, stomatal conductance, CO_2_ and water vapour concentrations. For each foliage sample, data collections to build the *A–C*_i_*–T* curves were completed within two or three consecutive days. Most of the *A–C*_i_ curves at 35 and 40 °C measured in 2015 at the Watford and Deville sites failed to converge and estimates could not be obtained. All measured gas exchange were corrected based on the measured projected needles area (see below).

### Estimation of gas exchange parameters

The photosynthetic parameters (*V*_cmax_, *J*_max_ and *g*_m_) were estimated simultaneously by fitting the *A–C*_i_ curves with the non-rectangular hyperbola version of the biochemical model of C_3_ (Farquhar *et al.*, 1980) following Ethier and Livingston (2004). This method is based on the principle that mesophyll conductance (*g*_m_) is not infinite, which reduces the curvature of the *A–C*_i_ curve. The net assimilation rate (*A*_n_) is given by:

$$A_{n}=\min\left\{A_{c},A_{j}\right\}$$ (1)

with

$$A_{c}=V_{\text{cmax}}\frac{\left(C_{c}-\Gamma^{*}\right)}{C_{c}+K_{c}\left(1+O/K_{o}\right)}-R_{\text{day}}$$ (2)

$$A_{j}=J\frac{C_{c}-\Gamma^{*}}{4\left(C_{c}+2I^{*}\right)}-R_{\text{day}}$$ (3)

Cc=Ci−Angm (4)

$$J=\frac{\alpha Q}{\sqrt{1+{\left(\frac{\alpha Q}{J_{\text{max}}}\right)}^{2}}}$$ (5)

The combination of eqns (2) and (4) results in a quadratic equation for the Rubisco-limited net photosynthetic rate (*A*_c_), whose solution is the following positive root:

$$A_{c}=\frac{-b+\sqrt{b^{2}-4\text{ac}}}{2a}$$ (6)

where

$$a=\frac{-1}{g_{m}}$$

$$b=\frac{\left(V_{\text{cmax}}-R_{\text{day}}\right)}{g_{m}}+C_{i}+K_{c}\left(1+\frac{O}{K_{o}}\right)$$

$$c=R_{\text{day}}\left[C_{i}+K_{c}\left(1+\frac{O}{K_{o}}\right)\right]-V_{\text{cmax}}\left(C_{i}-T^{*}\right)$$

The combination of eqns (3) and (4) results in a quadratic equation for the ribulose bisphosphate (RuBP) regeneration-limited net assimilation rate (*A*_j_), whose solution is the following positive root:

$$A_{j}=\frac{-b+\sqrt{b^{2}-4\text{ac}}}{2a}$$ (7)

where

$$a=\frac{-1}{g_{m}}$$

b=(J4−Rday)gm+Ci+2T*

$$c=R_{\text{day}}\left(C_{i}+2T^{*}\right)-\frac{J}{4}\left(C_{i}-T^{*}\right)$$

where *V*_cmax_ is the maximum rate of carboxylation (*µ*mol CO_2_ m^−2^ s^−1^), *O* is the partial atmospheric pressure of O_2_ (mmol mol^−1^)_,_Γ* is the CO_2_ compensation point in the absence of mitochondrial respiration, *R*_day_ is mitochondrial respiration in the light (*µ*mol CO_2_ m^−2^ s^−1^), *C*_i_ is the intercellular concentration of CO_2_ (µmol mol^−1^), *C*_c_ is the chloroplastic concentration of CO_2_ (µmol mol^−1^), *K*_c_ (µmol mol^−1^) and *K*_o_ (mmol mol^−1^) are the Michaelis–Menten constants of Rubisco for CO_2_ and O_2,_ respectively, *J* is the rate of electron transport (*µ*mol CO_2_ m^−2^ s^−1^), *J*_max_ is the maximum rate of electron transport (*µ*mol CO_2_ m^−2^ s^−1^), *Q* is the incident PAR (µmol m^−2^ s^−1^), *α* is the quantum efficiency, which represents the initial slope of the photosynthetic light response curve, and *g*_m_ is mesophyll conductance (mol CO_2_ m^−2^ s^−1^).

The model was fitted using non-linear regression techniques (Proc NLIN, SAS). To fit the model, the measured dark respiration (*R*_d_) values were used as proxy for *R*_day_ in order to reduce the number of parameters estimated by the model. Respiration occurring in daylight (*R*_day_), which is assumed to be primarily mitochondrial respiration, was assumed to approximate dark respiration (*R*_d_), as observed for black spruce by Way and Sage (2008*b*). The values at 25 °C used for *K*_c_ and *K*_o_ were 272 µmol mol^−1^ and 166 mmol mol^−1^, respectively (Sharkey *et al.*, 2007), and its temperature dependency is given by:

$$\text{Parameter}=e^{\left(c-\frac{H_{a}}{\text{RT}}\right)}$$ (8)

where *c* is a scaling constant, *H*_a_ (kJ mol^−1^), is the activation energy and *R* is the universal gas constant. The scaling constant (*c*) values are 35.98, 12.37 and 11.18 for *K*_c_, *K*_o_ and Γ*, respectively. The values of *H*_a_ are 80.99, 23.72 and 24.46 for *K*_c_, *K*_o_ and Γ*, respectively (Sharkey *et al.*, 2007).

Characterization of the temperature responses of gas exchange parameters

Individual photosynthesis temperature response curves (*A*_n_*–T*) were fitted with a quadratic model (eqn 9) as described by Battaglia *et al.* (1996):

$$A_{n}\left(T\right)=A_{\text{opt}}-b{\left(T-T_{\text{opt}}\right)}^{2}$$ (9)

where *A*_n_(*T*) is the photosynthetic rate at temperature *T* in °C, *A*_opt_ is the photosynthetic rate at the temperature optimum (*T*_opt_) and the parameter *b* describes the curvature of the parabola.

Dark respiration temperature response curves (*R*_d_–*T*) were analysed using eqn (10) to estimate *Q*_10_, which is the change in respiration with a 10 °C increase in temperature, following Atkin *et al.* (2005):

$$R_{d}\left(T\right)=R_{d}{}_{10}Q_{10}{}^{\left[(T-10)/10\right]}$$ (10)

where *R*_d_ (*T*) is the dark respiration rate at temperature *T* in °C and *R*_d10_ is the measured rate of *R*_d_ at the reference temperature of 10 °C.

Both *J*_max_*–T* and *V*_cmax_*–T* data showed a deactivation at high temperatures. Thereafter, the response of *V*_cmax_ and *J*_max_ to needle temperature were fitted using a modified Arrhenius function (the peaked model) (Johnson *et al.*, 1942) following Medlyn *et al.* (2002):

$$K\left(T_{k}\right)=K_{25}\frac{e^{\left[\frac{H_{a}}{RT_{\text{ref}}}\left(1-\frac{T_{\text{ref}}}{T_{k}}\right)\right]}}{1+e^{\left[\frac{\Delta ST_{k}-H_{a}}{RT_{k}}\right]}}\left[1+e^{\left(\frac{\Delta ST_{\text{ref}}-H_{d}}{RT_{r\text{ef}}}\right)}\right]$$ (11)

where *K*(*T*_k_) is the *V*_cmax_ or *J*_max_ at temperature *T*_k_ which is the leaf temperature in Kelvin, *K*_25_ is the value of *V*_cmax_ or *J*_max_ at *T*_*re*f_ = 25 °C, *R* is the universal gas constant (8.314 J mol^−1^ K^−1^), *H*_a_ (kJ mol^−1^) is the activation energy, *H*_d_ (kJ mol^−1^) is the energy of deactivation and *ΔS* (J mol^−1^) is an entropy term.

The *T*_opt_ for *V*_cmax_ and *J*_max_ was calculated as:

Topt=HdΔS−Rln[Ha(Hd−Ha)] (12)

The model was fitted using non-linear regression techniques (Proc NLIN, SAS). The value of *H*_d_ was fixed to 200 kJmol^−1^ according to Medlyn *et al.* (2002) in order to reduce the number of parameters estimated by the model. However, the model underestimates the value of *T*_opt_. Consequently, we first estimated *T*_opt_ using eqn (9) and then used the obtained value to solve for *H*_a_ and *ΔS* using eqns (11) and (12) simultaneously.

Mesophyll conductance was modelled according to the empirical model proposed by Warren and Dreyer (2006):

gm=gopte[−0.5[ln(TTopt)/b]2] (13)

where *g*_m_ is the mesophyll conductance, *g*_opt_ is the value of *g*_m_ at *T*_opt_ and *b* is a scaling factor.

### Quantitative limitation analysis

Rubisco-limited (*A*_c_) and RuBP regeneration-limited (*A*_j_) net photosynthetic rates (*A*_n_) were calculated at temperature ranging from 10 to 40 °C using eqns (2)–(5). Values of *V*_cmax_ and *J* were estimated from fitted parameters in our study. *R*_day_ was assumed to approximate *R*_d_ (see above).

### Needle nitrogen concentrations (*N*_mass_) and specific leaf area

Following the measurements of gas exchange, the shoots were carefully removed from the cuvette, harvested, placed in plastic bags and refrigerated (−20 °C). Projected needle area was measured using WinSeedle (Version 2007 Pro, Regent Instruments, Québec, Canada). Samples were then oven-dried for 72 h at 56 °C and their dry mass was determined. Specific leaf area (SLA) was calculated as the ratio of projected needle area (cm^2^) to needle dry mass (g). Dried needles were ground to a fine powder in a ball mill. Needle nitrogen concentration (*N*_mass_, mg g^−1^) was determined using a LECO elemental analyser (LECO Corporation, St Joseph, MI, USA). Nitrogen on a projected area basis (*N*_area_) was calculated as *N*_mass_ divided by SLA.

### Statistical analyses

All analyses were conducted with SAS/STAT software version 9.4 (SAS Institute, Cary, NC, USA). Response variables were analysed separately using a general linear mixed model with the effects of site and seed source (seed orchard) and their interaction considered as fixed effects, while block was treated as a random effect. Data were transformed whenever required to satisfy normality of residuals and homoscedasticity. Means were compared using the Tukey test; differences were considered significant at *P* < 0.05. We compared *H*_a_, *ΔS* and *T*_opt_ for *V*_cmax_, *J*_max_ and *g*_m_ between the two seed sources by non-parametric tests using proc NPAR1WAY. Proc REG and proc NLIN were used to examine the relationship between response variables and climate of plantation sites. The lack of *A–C*_i_ curves at 35 and 40 °C from measurements at the Watford and Deville sites in 2015 made it difficult to examine the site effect on *H*_a_ and *T*_opt_ of *V*_cmax_ and *J*_max_. To overcome this problem, we used a repeated-measures analysis of variance (ANOVA) with temperature from 10 to 30 °C as a repeated measure factor.

## RESULTS

### Growth

**Fig. 2.**
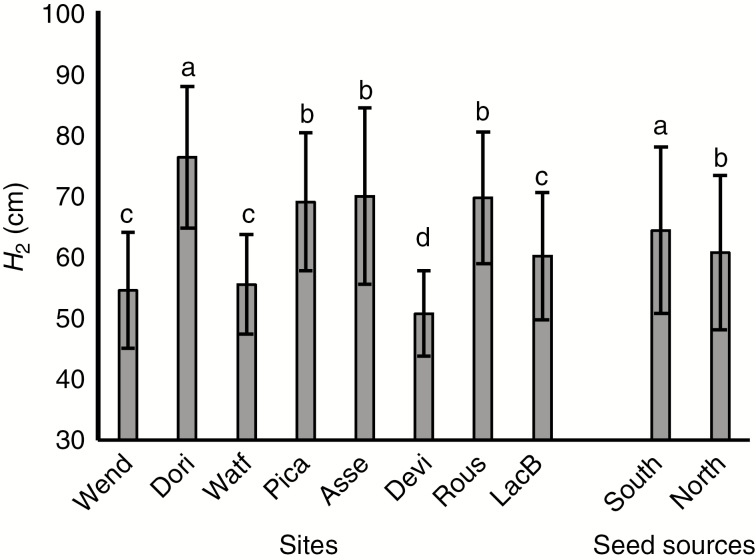
Total height (mean ± s.d.) at the end of the second growing season (*H*_2_) of white spruce seedlings from two seed sources grown at eight forest plantation sites. Means having the same letters are not significantly different at α = 0.05. Wend, Wendover; Dori, Dorion; Watf, Watford; Pica, Picard; Asse, Asselin; Devi, Deville; Rous, Rousseau; LacB, Lac Bergeron; South, southern seed source (SO1-1); north, northern seed source (SO1-5).

Total height growth after two growing seasons (*H*_2_) was affected by both site and seed source effects (Table 2). However, the interaction between the two factors was not significant, suggesting that the two seed sources showed similar patterns of growth in response to changes in growing conditions along the climatic gradient (Table 2). The highest and lowest average of *H*_2_ were observed at the Dorion (76 ± 11 cm) and Deville (51 ± 7 cm) sites, respectively (Fig. 2). Seedlings from the southern seed source were significantly taller than those from the northern seed source (Fig. 2). No statistically significant relationships could be found between *H*_2_ and prevailing climatic and soil conditions at the plantation sites (Supplementary Data Fig. S1).

**Table 2.** Analysis of variance of height growth after two growing seasons (H_2_) and thermal acclimation-related traits

	Site			Seed source			Site × seed source		
	d.f.	*F*	*P* value	d.f.	*F*	*P* value	d.f.	*F*	*P* value
*H*_2_	7	84.57	**<0.001**	1	32.76	**<0.001**	7	1.34	0.25
*T*_opt_ (*A*_n_)	6	1.61	0.19	1	0.22	0.64	6	1.43	0.24
*A*_opt_ (*A*_n_)	6	6.11	**<.001**	1	3.91	0.06	6	2.46	0.06
*T*_opt_ (*A*_g_)	6	1.03	0.43	1	1.65	0.21	6	1.86	0.13
*g*_s___25_	6	6.6	**<0.001**	1	1.19	0.29	6	1.27	0.31
*Q*_10_	7	3.45	**<0.001**	1	0.26	0.61	6	1.21	0.32
*R*_d10_	7	6.21	**<0.001**	1	1.09	0.31	6	0.94	0.48
*N*_mass_	7	2.93	**0.01**	1	0.2	0.65	6	1.22	0.32
SLA	7	2.22	0.06	1	2.85	0.10	7	0.51	0.79

*H*_2_ (cm), height growth after two growing seasons; *T*_opt_ (°C), optimal temperature for net (*A*_n_) and gross (*A*_g_) photosynthetic rate; *A*_opt_ (µmol m^−2^ s^−1^), photosynthetic rate at *T*_opt_. *g*_s___25_, stomatal conductance at a reference temperature of 25°C; *Q*_10_, rate of change in dark respiration with a 10 °C increase in temperature; *R*_d10_ (µmol m^−2^ s^−1^), basal rate of dark respiration (at 10 °C); *N*_mass_, needle nitrogen concentration (mg g^−1^); SLA, specific leaf area (cm^2^ g^−1^).

Significant effects are indicated in bold.

### Latitudinal variation in temperature response of photosynthesis and respiration

**Fig. 3.**
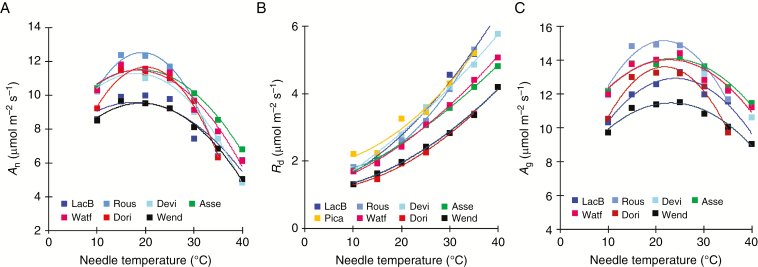
Temperature response curves of (A) net photosynthesis (*A*_n_), (B) dark respiration (*R*_d_), and (C) gross photosynthesis (*A*_g_) of two geographically distant white spruce seed sources grown in eight plantation sites (*n* = 6). For site abbreviations see legend of Fig. 2. At Picard (Pica), the measurements were limited to *R*_d_ and the northern seed source.

**Fig. 4.**
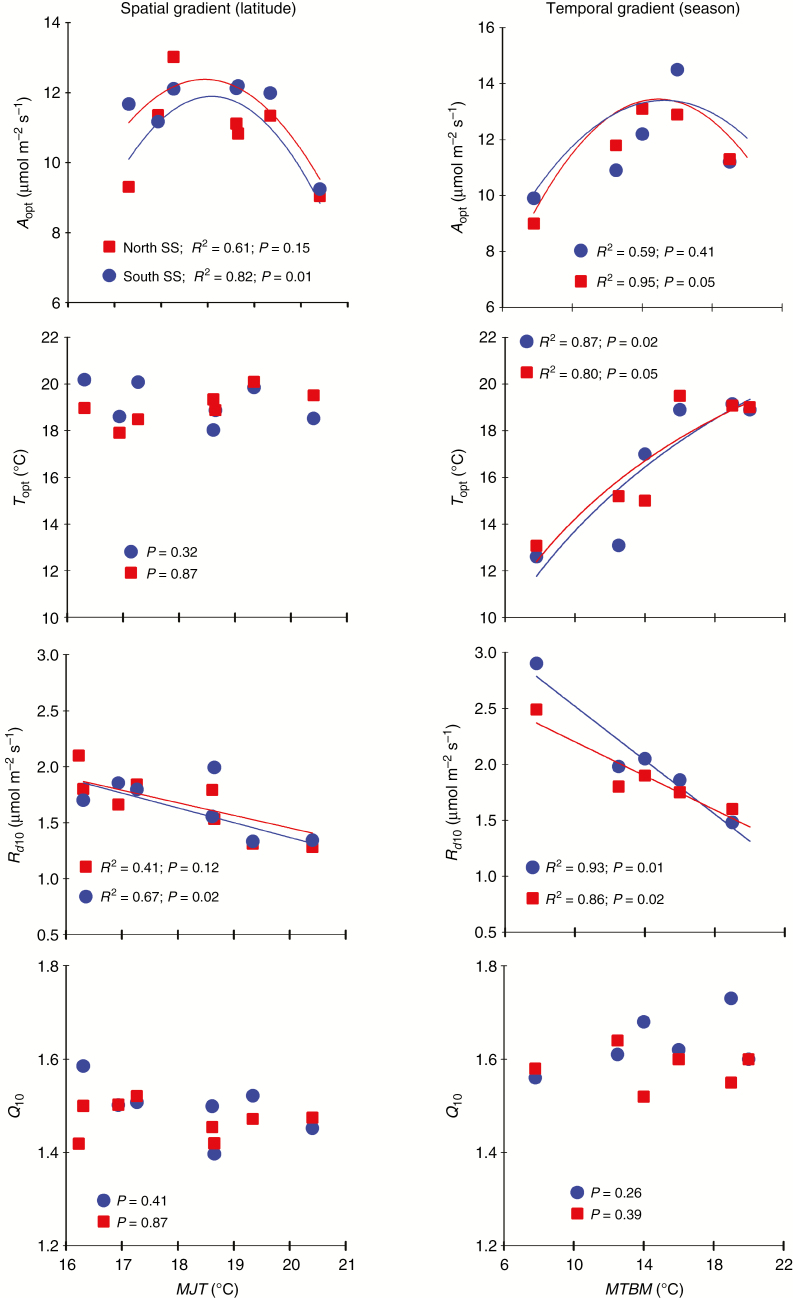
Relationships of thermal acclimation-related traits with climatic conditions along a latitudinal gradient (spatial) and during a growing season (temporal gradient) for two geographically distant white spruce seed sources. MJT, mean July temperature of plantation site during the two growing seasons (1 year before and current year of measurement); MTBM, mean temperature 5 d before measurements; *T*_opt_, optimal temperature for net photosynthetic rate; *A*_opt_, photosynthetic rate at *T*_opt_; *R*_d10_, basal rate of respiration (*T* = 10 °C); *Q*_10_, rate of change in *R*_d_ with a 10 °C increase in temperature. South SS, southern seed source (SO1-1); North SS, northern seed source (SO1-5).

The temperature response curves of both net and gross photosynthesis followed a parabolic shape (Fig. 3). The thermal optimum (*T*_opt_) of net photosynthesis (*A*_n_) averaged 19 ± 1.2 °C and was not variable across plantation sites and seed sources (Tables 2 and 3). Net photosynthetic rate (*A*_opt_) at *T*_opt_ varied significantly among sites (Table 2), with the lowest value of *A*_opt_ occurring at the Wendover and Lac Bergeron sites, which were the warmest and coldest sites, respectively (Table 3). *A*_opt_ followed a quadratic relationship with mean July temperature of the plantation site (MJT) for the southern seed source but not for the northern one (Fig. 4). Basal rate of *R*_d_ (*R*_d10_) and *Q*_10_ showed similar responses among seed sources but differed across plantation sites (Table 2). The lowest mean values of *R*_d10_ were measured at the warmest sites, such as Wendover and Dorion (Table 3), while the highest mean values of *Q*_10_ occurred at Lac Bergeron, the coldest plantation site (Table 3). *T*_opt_ and *Q*_10_ of dark respiration were not related to climatic variables at the plantation sites (Fig. 4). *R*_d10_ was negatively and linearly related to MJT for the southern seed source (Fig. 4). The temperature response curve of gross photosynthesis (*A*_g_) showed a shape similar to that of *A*_n_ (Fig. 3). The average *T*_opt_ of *A*_g_, 22.7 ± 1.3 °C, was not affected by either site or seed source (Table 2).

**Table 3.** Means (± s.d., *n* = 6) of thermal acclimation-related traits for photosynthesis and dark respiration of two geographically distant white spruce seed sources established along a latitudinal gradient of eight forest plantation sites

Plantation site	*A*_opt_(µmol m^−2^ s^−1^)	*T*_opt_(°C)	*R*_d10_(µmol m^−2^ s^−1^)	*Q*_10_
Lac Bergeron	10.0 (1.5)^b^	19.6 (1.1)^a^	1.7 (0.4)^a^	1.56 (0.06)^a^
Rousseau	12.6 (1.5)^a^	19.3 (1.1)^a^	1.8 (0.5)^a^	1.52 (0.10)^ab^
Deville	11.2 (0.4)^a^	18.6 (1.1)^a^	1.8 (0.3)^a^	1.50 (0.03)^b^
Asselin	11.5 (1.4)^a^	18.9 (0.8)^a^	1.7 (0.3)^a^	1.42 (0.04)^d^
Picard	–	–	2.2 (0.6)^a*^	1.42 (0.10)^d^*
Dorion	11.7 (0.9)^a^	19.9 (0.5)^a^	1.3 (0.3)^b^	1.47 (0.10)^c^
Watford	11.6 (1.4)^a^	18.7 (1.3)^a^	1.7 (0.4)^a^	1.45 (0.05)^cd^
Wendover	9.6 (1.4)^b^	19.1 (1.6)^a^	1.3 (0.5)^b^	1.48 (0.09)^c^

*T*_opt_, optimal temperature for net (*A*_n_) photosynthetic rate; *A*_opt_, photosynthetic rate at *T*_opt_; *Q*_10_, rate of change in dark respiration with a 10 °C increase in temperature; *R*_d10_, basal rate of dark respiration (at 10 °C).

Within columns, means followed by the same letter do not differ significantly at α = 0.05 based on adjusted Tukey’s tests.

Measurements were carried out during the second growing season in Lac Bergeron, Rousseau, Dorion and Wendover and during the third growing season in other sites.

Plantation sites are ordered from north to south.

*For the Picard site, measurements were limited to the northern seed source.

### Seasonal variation in temperature response of photosynthesis

**Fig. 5.**
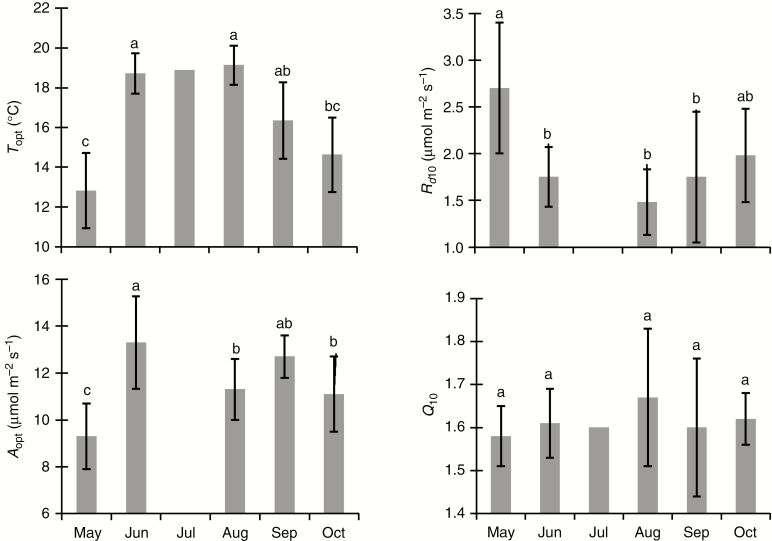
Seasonal patterns of thermal acclimation-related traits of two geographically distant white spruce seed sources during the 2016 growing season in the Watford plantation site. Values are mean ± s.d, *n* = 6. Values for July are not included in the anlaysis because they are from other seedlings (those used for *A–C*_i_ curve measurements). Means having the same letters are not significantly different α = 0.05. For abbreviations see legend of Fig. 4.

Thermal optimum (*T*_opt_) and *A*_opt_ of *A*_n_ varied greatly during the growing season (from May to October). *T*_opt_ and *A*_opt_ showed no interaction effect of seed source and month during the growing season (Table 4). Mean *T*_opt_ varied from 12.6 to 19.5 °C and was highest in June and August and lowest in May (Fig. 5). Mean *A*_opt_ was higher in June than in May (Fig. 5). Both *A*_opt_ for the northern seed source and *T*_opt_ for both seed sources were correlated with mean temperature 5 d before measurements (Fig. 4). The basal rate of *R*_d_ (*R*_d10_) but not *Q*_10_ varied greatly during the growing season and neither of them showed any interaction effect of seed source and month (Table 4). Mean *R*_d10_ was higher in May than in the remaining months excluding October (Fig. 5). *R*_d10_ was negatively and linearly related to mean temperature 5 d before measurements (Fig. 4). In contrast, *Q*_10_ was not related to mean temperature 5 d before measurements (Fig. 4).

**Table 4.** Analysis of variance of thermal acclimation traits during the 2016 growing season in the Watford plantation site

	Month			Seed source			Month × seed source		
	d.f.	*F*	*P* value	d.f.	*F*	*P* value	d.f.	*F*	*P* >value
*T*_opt_	4	12.5	**<0.001**	1	0.01	0.94	4	0.62	0.65
*A*_opt_	4	3.97	**0.02**	1	0.53	0.47	4	1.47	0.25
*Q*_10_	4	1.02	0.42	1	1.60	0.27	4	1.94	0.15
*R*_d10_	4	3.67	**0.03**	1	0.14	0.73	4	1.19	0.35

See Table 2 footnote for abbreviations.

Significant effects are indicated in bold.

### Temperature response of *V*_cmax_, *J*_max_ and *g*_m_

**Fig. 6.**
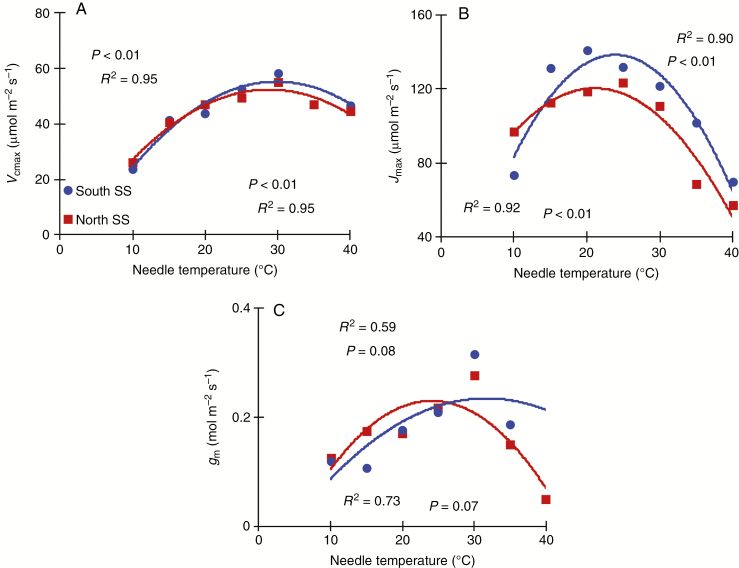
Temperature response curve of (A) the maximum carboxylation capacity of rubisco (*V*_cmax_), (B) the maximum electron transport rate (*J*_max_) and (C) mesophyll conductance to CO_2_ (*g*_m_) of two geographically distant white spruce seed sources measured in the Watford plantation site in 2016 (fourth growing season) (*n* = 3). South SS, southern seed source (SO1-1); North SS, northern seed source (SO1-5).

For the 2015 measurements (third growing season), repeated measures ANOVA showed that the temperature response curve of *V*_cmax_ and *J*_max_ (for temperatures between 10 and 30 °C) was not different between the southern (Watford) and northern (Deville) plantation sites (Table 5). The *J*_max25_:*V*_cmax25_ ratio averaged 2.5 ± 0.2 and was also not different between the two plantation sites and not different between the two seed sources. For the measurements conducted only in the Watford plantation site in 2016 (fourth growing season), the temperature response curve of *V*_cmax_, *J*_max_ and *g*_m_ displayed marked increases with temperature, followed by decreases above *T*_opt_ (Fig. 6). The optimal temperature (*T*_opt_) for *V*_cmax_ and *J*_max_ was higher for the southern seed source than for the northern one (Table 6). *T*_opt_ for *g*_m_ was similar for the two seed sources and averaged 28 ± 1.1 °C. The activation energy (*H*_a_) for *J*_max_ was greater for the southern seed source than for northern one but this was not the case for *V*_cmax_ (Table 6). Also, the entropy term of *V*_cmax_ was greater for the northern seed source than for the southern one (Table 6).

**Table 5.** Repeated-measures ANOVA of maximal rate of carboxylation (*V*_cmax_) and maximal rate of electron transport (*J*_max_) in response to needle temperature of two geographically distant white spruce seed sources during the third growing season in Watford and Deville plantation sites (respectively the easiest to access among southern and northern sites)

		*V*_cmax_		*J*_max_	
Effect	d.f.	*F*	*P* value	*F*	*P* value
Site	1	0	0.9858	0.77	0.4034
Seed source	1	0.16	0.7026	0.93	0.3608
Site × seed source	1	2.28	0.1651	1.82	0.2097
Temperature	4	62.91	**<0.0001**	16.14	**<0.0001**
Site × temperature	3	0.9	0.46	0.8	0.5057
Seed source × temperature	3	0.91	0.4787	0.22	0.9229
Site × seed source × temperature	3	0.67	0.5776	1.27	0.3089

Significant effects are indicated in bold.

**Table 6.** Means (± s.d., n = 3) of physiological parameters related to temperature response of needle photosynthetic capacity and mesophyll conductance of two white spruce seed sources measured during the fourth growing season at the Watford plantation site

		Southern seed source	Northern seed source
*V*_cmax_	*T*_opt_ (°C)	31.2 (4.1)^a^	28.1 (0.5)^b^
	Value at 25 °C (µmol m^−2^ s^−1^)	52.4 (7.5)^a^	52.3 (4.5)^a^
	*H*_a_ (kJ mol^−1^)	32.9 (9.2)^a^	26.7 (5)^a^
	*∆S* (J K^−1^ mol^−1^)	640.1 (7.1)^a^	661 (4.5)^b^
*J*_max_	*T*_opt_ (°C)	24.2 (3.2)^a^	20.9 (1.1)^b^
	Value at 25 °C (µmol m^−2^ s^−1^)	131.1 (24.3)^a^	122.8 (7.2)^a^
	*H*_a_ (kJ mol^−1^)	18.4 (10.1)^a^	14.6 (6.1)^b^
	*∆S* (J K^−1^ mol^−1^)	644.9 (8.9)^a^	649.2 (2.2)^a^
*g*_m_	*T*_opt_ (°C)	28.4 (1.4)^a^	28.7 (1.1)^a^
	Value at 25 °C (mol m^−2^ s^−1^)	0.22(0.04)^a^	0.21(0.07)^b^

*V*_cmax_, maximal rate of carboxylation; *J*_max_, maximal rate of electron transport; *g*_m_, mesophyll conductance; *H*_a_, activation energy, *∆S*, entropy term, *T*_opt_, optimal temperature.

Parameters were derived from eqn (11) for *V*_cmax_ and *J*_max_ and from eqn (13) for *g*_m_. Within rows, means followed by the same letter do not differ significantly at α = 0.05.

**Fig. 7.**
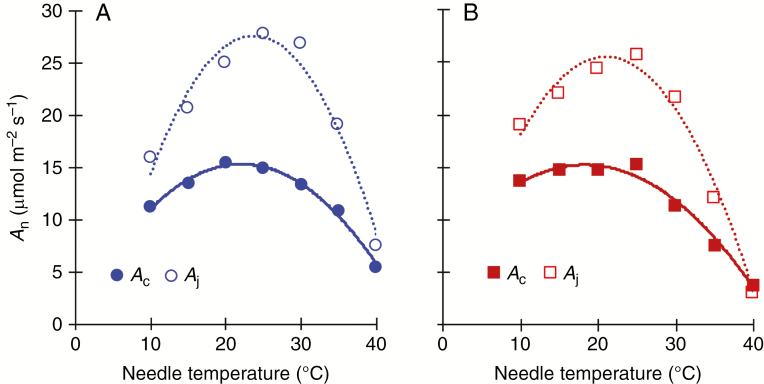
Biochemical limitations of *A*_n_ in response to needle temperature for (A) the southern seed source and (B) the northern seed source. *A*_n_ was modelled at *C*_a_= 400 ppm by Farquhar modelling using measured *g*_s_ and estimated *g*_m_ from *A*–*C*_i_. The response of *A*_n_ is defined by the minimum value of either Rubisco-limited (*A*_c_) (solid curve) or RuBP regeneration-limited (*A*_j_) net photosynthesis (dashed curve).

The temperature responses of Rubisco-limited (*A*_c_) and RuBP regeneration-limited (*A*_j_) net photosynthesis (*A*_n_) were similar among seed sources. Except at 40 °C, *A*_j_ was higher than *A*_c_ (Fig. 7). Consequently, changes in temperature dependence of photosynthesis were mainly *V*_cmax_-dependent for temperature range between 10 and 40 °C.

### Relationships between thermal acclimation-related traits, *N*_mass_ and SLA

Specific leaf area was not affected either by site or seed source (Table 2; Supplementary Data Fig. S2). In addition, it was not related to the climate of the plantation sites (MJT) (*P* ≥ 0.10). Needle nitrogen concentration (*N*_mass_) was similar among seed sources but was affected by sites (Table 2). Similar results were obtained using needle nitrogen content per unit of needle projected area. Mean *N*_mass_ was greater in the Asselin, Deville and Picard sites than in the Lac Bergeron, Wendover and Dorion sites (Supplementary Data Fig. S2). Both *R*_d10_ and *A*_opt_ were unrelated to leaf nitrogen (*N*_mass_) (Supplementary Data Fig. S3).

## DISCUSSION

Under future climate change, temperature will likely remain the most important factor driving boreal forest productivity and species distribution, with potential effects on the composition and structure of natural populations. However, thermal acclimation of physiological processes, particularly photosynthesis, could help mitigate the predicted decrease in net productivity associated with global warming (Way and Yamori, 2014; Girardin *et al.*, 2016). Consequently, a better understanding of acclimation of both photosynthesis (*A*_n_) and dark respiration (*R*_d_) to warming might provide valuable information to predict the capacity of species to adapt under climate change, and help improve the predictive accuracy of process-based models (Slot *et al.*, 2014; Lombardozzi *et al.*, 2015). Investigations of thermal acclimation of *A*_n_ and *R*_d_ in natural conditions are limited compared with those conducted under controlled conditions. Studies conducted along a natural latitudinal gradient should be valuable to investigate growth habit and the thermal acclimation capacity at the intraspecific level, as it takes into account the seasonal fluctuations in temperature (monthly and daily temperature range) as well as the complex interactions with other climatic and non-climatic factors. The present study provides an exhaustive assessment of thermal acclimation of photosynthesis and dark respiration for a conifer under natural conditions, with valuable insights into the temperature responses of photosynthetic capacity attributes (*V*_cmax_ and *J*_max_) and mesophyll conductance under natural conditions. The present results are original in clearly showing a lack of thermal acclimation of net photosynthesis (*A*_n_) and evidence for type II acclimation of dark respiration (*R*_d_) in response to long-term (2–4 years) variation in temperature along the regional climatic gradient of 5.5 °C for both the southern and northern white spruce seed sources tested.

Boreal tree species, which are well adapted to cold temperature, were often assumed to be limited in their capacity to adapt to warm conditions (Girardin *et al.*, 2016). We showed that the qualitative aspect of thermal acclimation of photosynthesis, such as the ability to shift the thermal optimum (*T*_opt_) of *A*_n_, was lacking. *T*_opt_ was similar along the climatic gradient and was close to the mean July temperature at southern plantation sites. Our results do not agree with those of previous studies on other species of *Picea* conducted in controlled conditions, which showed a shift in *T*_opt_ when the growing temperature was increased by 10 °C (Way and Sage, 2008*a*; Zhang *et al.*, 2015). Despite the variation in mean temperature (MAT and MJT), a similar range of temperatures (minimum and maximum) along the climatic gradient tested here may explain the unchanging *T*_opt_ of photosynthesis observed in our study. Increased or constant photosynthetic rate at *T*_opt_ towards a warmer environment has been widely used as a quantitative proxy of thermal acclimation of photosynthesis (Way and Yamori, 2014). In accordance with results of previous studies on other *Picea* species (Way and Sage, 2008*a*; Zhang *et al.*, 2015), we showed the inability of the two seed sources to maintain photosynthetic performance (*A*_opt_) in warmer sites. In addition, the relationship between *A*_opt_ and the growing temperature along the gradient followed a quadratic shape, which may suggest adaptation to a narrow climate niche. However, this pattern may also result from complex interaction between soil and climatic conditions (temperature and precipitation) along the climatic gradient tested here rather than temperature *per se* (Reich and Oleksyn, 2004; Chi *et al.*, 2013; Scafaro *et al.*, 2016). In our study, variation in leaf nitrogen content appeared to play a minor role in photosynthetic adjustments.

In the present study, the temperature response of *A*_n_ was Rubisco-limited (*V*_cmax_) over the entire range of needle temperature (10–40 °C), which follows the common trend observed for cold-adapted species (Kattge and Knorr, 2007; Sage and Kubien, 2007; Sage *et al.*, 2008; Yamori *et al.*, 2010). This result suggests that the observed lack of thermal acclimation potential of white spruce may result from (1) lack of nitrogen reallocation from *A*_j_ to *A*_c_ and (2) Rubisco activase (RCA) lability. The similar *J*_max25_:*V*_cmax25_ ratio between the southern and northern plantation sites is in accordance with our previous results during the second growing season at the same sites for six seed sources, including those used here (Benomar *et al.*, 2016), and with other reports (Sage *et al.*, 2008; Dillaway and Kruger, 2010). This lack of adjustment of nitrogen invested in Rubisco (and other soluble proteins involved in the Calvin cycle) may be due to cold adaptation-related constraints on nitrogen allocation. In fact, it has been reported that cold-adapted species allocate more nitrogen to *J*_max_ as a compensatory response to low temperature (Yamori *et al.*, 2010). *V*_cmax_ depends not only on Rubisco concentration but also on its activation state (inhibited/activated) (Salvucci and Crafts-Brandner, 2004; Sage *et al.*, 2008). The activation state of Rubisco is regulated by RCA, a heat-labile enzyme using energy via ATP hydrolysis to release inhibitors from the active site of Rubisco (Crafts-Brandner and Salvucci, 2000; Yamori and von Caemmerer, 2009). A decrease in RCA activity has been documented as a primary cause of reduced Rubisco activity and then photosynthetic performance in response to increasing growth temperature (Yamori and von Caemmerer, 2009). Investigations regarding genetic variation in RCA and its activity in response to temperature in white spruce would help refine our understanding of the observed biochemical photosynthetic responses to temperature.

Although a lack of thermal acclimation of photosynthesis seems to be common to all boreal tree species (Way and Sage, 2008*a*; Dillaway and Kruger, 2010), thermal acclimation of dark respiration was reported to be moderate to strong for several tree species of the boreal forest, including white spruce (Tjoelker *et al*., 1999, 2009; Dillaway and Kruger, 2011; Reich *et al.*, 2016; Wei *et al.*, 2017). It seems that the firm acclimation of *R*_d_ (predominantly by the downshift in *Q*_10_) could reduce the negative impact of rising temperatures on photosynthesis under climate change (Atkin *et al.*, 2005; Reich *et al.*, 2016). In accordance with the recent results of Reich *et al.* (2016) for white spruce and the results of Tjoelker *et al.* (1999) for black spruce, we showed evidence for type II acclimation of *R*_d_, as indicated by a downshift of *R*_d10_ with increasing plantation site temperature. However, we could not show consistent evidence for type I acclimation of *R*_d_ (Fig. 4) as in Reich *et al.* (2016), and Tjoelker *et al.* (1999) for different white spruce and black spruce seed sources. The average *Q*_10_ value (1.50 ± 0.15) observed in this study was similar to that obtained with a white spruce population from northern Minnesota, USA (Reich *et al.*, 2016). The small change noted in *Q*_10_ was not congruent with the important change in climate observed from site to site. Whether this lack of change in *Q*_10_ is related to the regional temperature gradient and seed sources tested in the present study, or to intrinsic species physiological performance needs to be examined (Wei *et al.*, 2017).

Needle nitrogen concentration had little impact on the thermal acclimation of *R*_d_, as indicated by similar results when *R*_d_ was expressed in nitrogen units. The observed type II acclimation of *R*_d_ might be a consequence of a change in needle mitochondria density or by mitochondrial overexpression of alternative oxidase (AOX) (Atkin *et al.*, 2005).

We observed thermal acclimation of both needle respiration and photosynthetic rate in response to seasonal variation in temperature (i.e. reduction in *R*_d10_ at higher temperatures and increase in *T*_opt_ and *A*_opt_ with increasing temperature). *A*_opt_ reached a maximum value in June and September, with a clear linear relationship with temperature as expressed in 5-d mean temperature windows. It is still unclear whether photosynthesis phenology relies on temperature or photoperiod cues (Busch *et al.*, 2007; Bauerle *et al.*, 2012; Stinziano and Way, 2017). Unfortunately, our results cannot help clarify this issue. In fact, the decrease in *A*_n_ from June to August may be related to a decline in photosynthesis following bud set or to the increase in mean temperature. On the other hand, the increase in *A*_n_ from August to September cannot be linked to photoperiod. The similar values of *T*_opt_ observed from June to August is in accordance to the pattern along the latitudinal gradient. The strong adjustment in *T*_opt_ to lower temperatures in May and October, which may relate to a change in the activation energy of *V*_cmax_, represents good evidence for higher acclimation of *A*_n_ to cold temperature in white spruce. The latter may explain the higher performance of seed sources from Québec in northern regions of Canada, such as Alberta (Lu *et al.*, 2014).

The temperature at the location of origin of seed sources (climate of origin) in our study had no effect on their thermal acclimation-related traits. This result may be interpreted as a lack of intraspecific genetic variation in the thermal acclimation of photosynthesis, as recently reported for *Eucalyptus tereticornis* seedlings grown under controlled conditions (Drake *et al.*, 2017). However, the limitations imposed by our experiment do not allow a clear conclusion to be drawn. In fact, the southern seed source experienced only a 1.7 °C warming in mean July temperature at the most southern plantation site of Wendover (Table 1). Consequently, we could formulate two hypotheses. First, despite a difference of 2 °C in mean July temperature of geographical origins between the two white spruce seed sources used in this study, their parents may have experienced historically a similar range of temperatures, which would explain the lack of difference in *T*_opt_ between them. Thus, it would be interesting to test other seed sources from more meridional origins and warmer local climates, such as from southern Ontario and the USA, to further test the existence of genetic differentiation in thermal acclimation of photosynthesis. Our second hypothesis relates to the warming conditions experienced by seed sources in the southern plantation sites, which are likely insufficient to detect intraspecific variation in the thermal acclimation of photosynthesis that would be related to local genetic adaptation. Thus, using more southern plantation sites or augmented warming conditions in controlled environments would be necessary to test this last hypothesis.

### Conclusions

Our results indicate that the photosynthesis of seedlings of two white spruce seed sources from southern and northern origins had a lower thermal optimum of photosynthesis and no ability to acclimate to warmer temperature. In contrast, the seedlings showed a clear acclimation of dark respiration by the downshift of the basal rate of *R*_d_. However, dark respiration acclimation was insufficient to counterbalance the low photosynthetic rate in warmer plantation sites. In addition, the temperature response of photosynthesis was limited by Rubisco capacity, which suggests an effect of Rubisco activase or a lack of adjustment of nitrogen allocation. The results highlight the need for more research on thermal responses of photosynthesis and its biochemical limitations, with particular emphasis on Rubisco activase and on understanding of the main cues of photosynthesis phenology in spruces and other boreal forest trees. Together with monitoring at a more mature stage, this should help evaluate the effect of predicted autumnal warming on the global trend of photosynthesis. Overall, our results on growth and thermal acclimation-related traits for photosynthesis and dark respiration suggest that white spruce populations from southern Québec are already above their thermal thresholds and will remain so under predicted climate warming.

## SUPPLEMENTARY DATA

Supplementary data are available online at: www.aob.oxfordjournals.org and consist of the following. Table S1: soil physico-chemical properties during the second growing season in the eight plantation sites; Figure S1: total height growth of two white spruce seed sources at the end of the second growing season (*H*_2_) plotted against site mean July temperature (MJT), soil total nitrogen and soil C:N ratio. Figure S2: *N*_mass_ and SLA of plants of two white spruce seed sources growing at eight plantation sites; Figure S3: *A*_opt_ and *R*_d10_ plotted against *N*_mass_ for the two white spruce seed sources; raw data on *A*_n_ response to *C*_i_ and temperature.

Supplementary Data
