# Supplementary material for: Thermal acclimation of photosynthesis and respiration of southern and northern white spruce seed sources tested along a regional climatic gradient indicates limited potential to cope with temperature warming
Source: Ann Bot. 2017 Dec 29;121(3):443–57. doi: 10.1093/aob/mcx174 (PMC5838847; doi:10.1093/aob/mcx174)
Supplement: Supplementary Data [file mcx174_suppl_supplementary_data.docx]

**SUPPLEMENTARY DATA**

Table S1: Soil physico-chemical properties during the second growing season in the eight plantation sites. Each value point is the mean of ten random soil cores (n=10).

| Site | *Total C* | *Total N* | *pH* | *P* | *K* | *CEC* | *NH4* | *Ca* | *Mg* | *Clay*  *%* | *Loam*  *%* | *Sand*  *%* |
| --- | --- | --- | --- | --- | --- | --- | --- | --- | --- | --- | --- | --- |
| Asselin | 47.8 | 2.6 | 4.1 | 11.7 | 60.1 | 34.1 | 4.2 | 703.9 | 43.7 | 24.6 | 37.1 | 38.3 |
| Deville | 43.2 | 2.2 | 3.8 | 11.6 | 61.8 | 36 | 6.1 | 134.1 | 25.8 | 23.9 | 26.9 | 49.2 |
| Dorion | 15.3 | 0.8 | 4.4 | 67.4 | 26.4 | 14.2 | 5.7 | 88.6 | 10.2 | 4.9 | 9.1 | 86.1 |
| Lac Bergeron | 30.2 | 1.2 | 4.4 | 4.8 | 10.7 | 28.5 | 3.9 | 28.8 | 5.2 | 9.7 | 17.5 | 72.8 |
| Picard | 40.4 | 1.9 | 4.3 | 7.7 | 27 | 32.2 | 3.8 | 97.4 | 12 | 10.3 | 31 | 58.8 |
| Rousseau | 13.7 | 0.9 | 4.3 | 6.8 | 181.7 | 24.6 | 6.5 | 1022.3 | 230.4 | 59.4 | 24.8 | 15.7 |
| Watford | 41.6 | 3.1 | 4.5 | 12.1 | 76.9 | 26 | 17.6 | 608.9 | 34.3 | 20 | 33.3 | 46.7 |
| Wendover | 22 | 0.9 | 4.8 | 14 | 24 | 13.8 | 2.4 | 350.1 | 34.8 | 6.9 | 9.6 | 83.5 |

**Fig. S1: Total height growth of two white spruce seed sources at the end of the second growing season (*H2*); plotted against site** mean July temperature **(*MJT*), soil total nitrogen and soil *C_N* ratio. South SS, southern seed source; North SS, northern seed source. The *P* value for curves was >0.1.**

Fig. S2**: Mean (±SD) *N_mass_* and *SLA* of plants of two white spruce seed sources growing at eight plantation sites.** Means having the same letters are not significantly different at α = 0.05. (n=6). Asse, Asselin; Devi, Deville; Dori, Dorion; LacB, Lac Bergeron; Pica, Picard; Rous, Rousseau; Watf, Watford; Wend, Wendover.

Fig. S3**: *A_opt_* and *Rd_10_* plotted against *N_mass_* for the two white spruce seed sources.**

**South SS, southern seed source; North SS, northern seed source. The *P* value for curves was >0.1.**
